# Expanding forest research with terrestrial LiDAR technology

**DOI:** 10.1038/s41467-025-63946-6

**Published:** 2025-10-06

**Authors:** Eduardo Eiji Maeda, Benjamin Brede, Kim Calders, Mathias Disney, Martin Herold, Emily R. Lines, Matheus Henrique Nunes, Pasi Raumonen, Miina Rautiainen, Ninni Saarinen, Iris Starck, Yanjun Su, Jonathan Terschanski, Louise Terryn

**Affiliations:** 1 Department of Geosciences and Geography, University of Helsinki, Helsinki, Finland; 2 Finnish Meteorological Institute (FMI), Helsinki, Finland; 3 GFZ Helmholtz Centre for Geosciences, Potsdam, Germany; 4 Q-ForestLab, Department of Environment, Faculty of Bioscience Engineering, Ghent University, Ghent, Belgium; 5 Department of Geography, University College London, London, UK; 6 NERC National Centre for Earth Observation, UCL Geography, London, UK; 7 Department of Geography, University of Cambridge, Cambridge, UK; 8 Department of Geographical Sciences, University of Maryland, College Park, MD USA; 9 Mathematics Research Centre, Faculty of Information Technology and Communication Sciences, Tampere University, Tampere, Finland; 10 School of Engineering, Aalto University, Espoo, Finland; 11 School of Forest Sciences, University of Eastern Finland, Joensuu, Finland; 12 Helsinki Institute of Sustainability Science, University of Helsinki, Helsinki, Finland; 13 Key Laboratory of Vegetation and Environmental Change, Institute of Botany, Chinese Academy of Sciences, Beijing, China; 14 China National Botanical Garden, Beijing, China

**Keywords:** Optical techniques, Forest ecology

## Abstract

The three-dimensional arrangement of plant components, both within and among individual trees, is fundamental for characterizing forest ecosystems. This structure not only influences but also responds to environmental changes, playing a key role in regulating light regimes, forest productivity, as well as physiological and biophysical processes. Over the past few decades, terrestrial laser scanning (TLS, or terrestrial LiDAR) has provided a unique perspective of this 3D structure, offering new insights into ecological processes and forest disturbances, as well as enhancing structural assessments in forest and carbon inventories. Here, we examine recent advancements in TLS and its applications in forest science. We also explore how increasing computational power, alongside the rise of artificial intelligence, is empowering researchers to tackle more complex questions, paving the way for breakthroughs in understanding forest ecosystem dynamics in a changing world.

## Abstract (web summary)

This Review highlights how terrestrial laser scanning is transforming forest research by enabling highly detailed 3D measurements of trees, supporting applications in forest ecology, carbon monitoring, and biodiversity assessment.

## Introduction

Remote sensing has revolutionized our understanding of forests worldwide. Since the first Earth observation missions were launched in the 1970s, remote sensing has contributed to quantifying and monitoring forest extent^1,2^, as well as forest functioning^3^. From tropical to boreal forests, these technologies have enabled us to track habitat loss^4^, ecosystem health^5^, and carbon storage^6^ at unprecedented spatial coverage and analytical depth, becoming an essential tool in monitoring key planetary boundaries related to land system change and climate regulation^7,8^.

Over the past decades, the field of forest remote sensing has made fast advancements in both methodological and computational capabilities. The field has shifted from simply mapping landscapes to actively monitoring dynamic processes at increasingly near real-time^9^. The ability to analyze the physiological and structural characteristics of forests has rapidly become both technically and operationally feasible. Light Detection and Ranging (LiDAR, also known as laser scanning) sensors mounted on aerial platforms have added a new dimension to these capabilities. By emitting laser pulses to measure distances, LiDAR technology provides precise and accurate, three-dimensional measurements of forest canopies. Although the first applications of airborne laser scanning (ALS) in forest studies began in the 1980s^10,11^, its widespread adoption did not gain momentum until after the 2000s^12^. Since then, LiDAR has become the remote sensing gold standard for quantifying forest structural characteristics such as canopy height and cover, and topographical features of the forest floor.

Among the remote sensing tools available to scientists, none provides a more accurate and detailed view of forest structure than terrestrial LiDAR technology, also known as terrestrial laser scanning (TLS). Unlike airborne systems, TLS instruments are positioned at ground level, allowing them to capture detailed measurements of both the forest understory and the upper canopy. Compared to other ground-based methods, such as mobile laser scanning, terrestrial photogrammetry, or traditional inventory techniques, TLS offers superior geometric accuracy and structural completeness, particularly for detailed modeling of individual trees and stand structure.

The adoption of TLS is much more recent in comparison to other remote sensing tools, increasing quickly from around 2010 onwards (Table 1). The first review articles on TLS applications in forests appeared in 2011, offering foundational insights into using TLS to assess forest structure, including tree height, stem diameter, and biomass^13^. Subsequent reviews highlighted TLS’s ability to improve plot-scale forest measurements and estimate tree metrics^14^, as well as its broader applications across forest science disciplines^15^. More recent reviews have summarized key developments and future challenges in TLS, reflecting the growing and diversifying TLS research community^16,17^. However, with the fast adoption of TLS for studying vegetation, substantial progress has been made beyond these latest assessments.

**Table 1:** Technical advancements and milestones in the development of terrestrial LiDAR (TLS) technology

Timeline	Milestones
1930s	Use of light pulses to measure distances, such as cloud heights, was pioneered by E.H. Synge^132^.
1960s	First lidar prototypes and commercial devices developed, driven by Theodore Maiman’s 1960 Ruby Laser invention at Hughes Research Laboratory, enabling high-precision range measurements and broad applications^133^.
1970s	Lidar expands into topographic and military applications^134^.
1980s	Integration of GPS with LiDAR boosts its use in mapping large areas, including airborne LiDAR applications.
1990s	Deployment of the first tripod-mounted 3D scanners (e.g., Cyra Technologies, later acquired by Leica), sparking advancements in terrestrial applications, and paving the way for broader adoption^135^.
2000s	First TLS applications in forest science emerge, focusing on tree height, stem diameter, and canopy structure^13^.
2010s	Rapid growth of TLS in forest studies, including biomass estimation, structural complexity^14,15^.
2020s	Advanced TLS techniques reveal temporal dynamics, improve canopy models, and enable global 4D monitoring initiatives^17,131^.

Although many factors have contributed to the uptake of TLS in forest studies, three aspects stand out: price, speed, and size. While high-end TLS instruments—typically characterized by high ranging accuracy and long effective range—remain prohibitively expensive for many research groups, the availability of more affordable devices has substantially increased in recent years, making the technology more accessible. Recent instruments are not only lighter but also offer significantly faster point acquisition rates. In addition, modern scanning protocols have become more efficient, as many systems no longer require fixed calibration targets for registration, reducing setup time and enabling faster fieldwork workflows^18^. These improvements allow researchers to cover greater areas more rapidly with multiple scan positions, which helps reduce occlusion and improve the completeness of forest structural data.

While increased accessibility and hardware improvements to TLS instruments have reduced or eliminated major data collection bottlenecks, extracting accurate information from the resulting point clouds remains a significant challenge. Recent algorithmic advances, including co-registration methods^19^, deep learning approaches for crown delineation^20^, and automated pipelines for large-scale tree extraction^21^, are streamlining TLS data processing and enabling more efficient analysis of complex point clouds. Together, these developments are expanding the scope of ecological research and transforming how we study forest structure and dynamics. This review provides a forward-looking assessment of the use of TLS technology in forest studies. We outline advancements made over the past years, explore emerging questions that TLS has the potential to address, and highlight the key challenges and bottlenecks that still limit its broader adoption and application.

## TLS and increasing realism in modeling forests

The rapid increase in our ability to use TLS to capture extremely detailed 3D descriptions of tree and forest structure has led to an increasing interest in so-called “digital twin” or virtual forest approaches^16,22^. But what does this mean in practice: do digital twins represent a useful new conceptual framework, a rebadging of “a model” or somewhere in between? There is no doubt that the concept of digital twins is being used to underpin some very large initiatives in linking climate, observation, and modeling (https://destination-earth.eu/).

According to Batty^23^ the term digital twin was coined in the early 2000s by Michael Grieves^24^ and has subsequently been used in a range of contexts. In its original sense, a digital twin represents a digital mirror image of a physical process, designed to match it precisely in both space and time, with a bidirectional flow of data between the physical and digital counterparts. A model, on the other hand, is generally considered as an abstraction of a physical process, keeping the key elements we are interested in, but simplifying or even ignoring those we are not. A model is therefore a simplified representation of a physical system, whereas a digital twin implies a representation with the maximum detail we can provide^23^.

A good example of where TLS has facilitated this distinction between a model and a digital twin is in the process of representing radiative transfer in vegetation^22,25^. This is a crucial application for quantifying and understanding the processes affecting canopy photosynthesis, modeling the Earth’s radiation budget, and biophysical feedback between vegetation and climate^26^. A great deal of work has gone into developing simplified radiative transfer models of vegetation for successful global monitoring of vegetation properties^27,28^, using a range of approximations to represent, for example, leaf and soil scattering properties, leaf amount, and physical arrangements in space. However, an alternative approach has been to represent the 3D physical canopy as accurately as possible, including every leaf or needle, branch, and soil element in 3D, and then solving the radiative transfer problem using, for example, Monte Carlo ray tracing (MCRT)^29,30^.

**Fig. 1:**
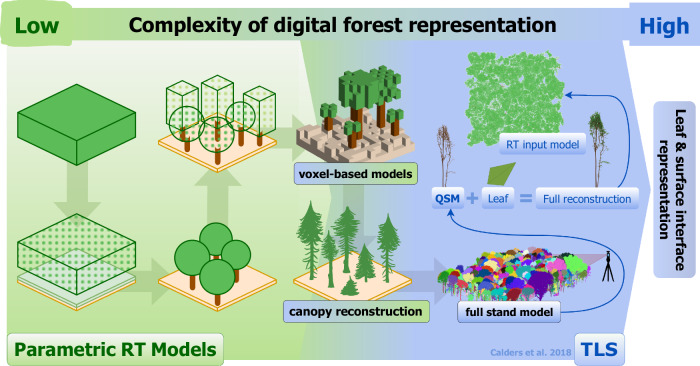
Progression of digital forest representation complexity, from simplified radiative transfer models to highly detailed reconstructions enabled by terrestrial laser scanning (TLS). On the left, parametric radiative transfer models depict forests as simple geometric shapes or approximations. In contrast, TLS-based reconstructions (right) provide high-resolution models of forest stands, including individual tree structures, detailed branching systems through quantitative structure models (QSMs), and foliage elements. TLS instruments capture dense 3D point clouds by scanning forests from multiple ground-based positions. These point clouds can then be processed to reconstruct tree geometry, crown shapes, and canopy surface details, supporting realistic structural inputs for modeling frameworks. The integration of TLS data into advanced modeling frameworks supports full forest reconstructions with applications in radiative transfer simulations, enhancing the realism of digital twins for forest ecosystems.

A limitation in this high-detail MCRT approach, however, is that it requires the spectral properties of every canopy element, including leaves, bark, and soil, to be known. In practice, acquiring such detailed measurements for every forest is not feasible, which prevents the use of these models for inversion—that is, retrieving canopy properties from remotely sensed reflectance. Between simplified parametric models and full 3D reconstructions, intermediate approaches like voxel-based representations (used in models, e.g., DART^31^) offer a balance between structural realism and computational efficiency, and can be parameterized with TLS data. Thus, rather than serving in large-scale monitoring applications, radiative transfer models based on TLS data help create a detailed scientific understanding of how forest structure influences multi-angular scattering processes of forest canopies^22^. TLS data can also broaden the scope for modeling canopy scattering, such as estimating photon recollision probability within forest canopies^32^. The progression of canopy realism in radiative transfer is illustrated in Fig. 1.

Another area where TLS is enabling this progression from simplified models to digital twins is in functional structural plant modeling (FSPM). The FSPM approach seeks to model the external structural expression of underlying genetic and phenotypic behavior^33,34^. FSPMs predict the 3D plant structure that arises from these underlying behaviors. The difficulty is testing and validating these structural predictions for real trees. There have been various attempts to couple 3D tree structure with FSPMs via manual measurements and even procedural models^33,35^. However, the advancements in TLS have opened the way to a much more effective parameterization of FSPMs, as well as direct tests of their predictions. O’Sullivan et al.^34^ suggested that TLS will contribute to FSPM development in two key areas: first, by parameterizing static FSPMs to simulate interactions between structure, environment, and physiology; and second, by enabling the testing and calibration of dynamic FSPM predictions to explore ecological and environmental hypotheses. Potapov et al.^36^, for example, developed a stochastic version of an existing FSPM (LIGNUM) for producing tree structures consistent with detailed TLS data. Sievänen et al.^37^ used TLS measurements of pine trees of different ages to construct a pseudo-time series of growth of a single tree. They used an FSPM to stochastically simulate crown development to match the TLS-derived crown development and suggested the resulting best-matching FSPM parameters represent the underlying crown development mechanisms. This ability to establish quantitative links between structure and function has enabled the development of a so-called structural economics spectrum, embedding tree size and structural diversity in the wider framework of plant resource use^38^.

Advances in capturing tree and forest structure via TLS are enabling the transition from simplified structural representations to digital twins, with very high levels of structural detail, using so-called quantitative structure models (QSMs), the algorithmic enclosure of point clouds in topologically-connected, closed volumes^39,40^. This, in turn, throws up some interesting challenges in terms of how best to use or interpret this detail. In radiative transfer modeling, for example, the challenge is no longer one of representing structure, but how to assign the underlying scattering properties of that structure—the leaves, branches, soil, etc. that make up the resulting scene model. This process will look very different across different wavelength domains, from the shortwave visible to thermal and microwave. In the case of FSPMs, a challenge will be how to feed back the phenotypic information expressed in observed structure to the underlying functional process representation. Challenges of course open further opportunities.

Returning to the question of whether digital twins represent something new and useful for forest monitoring, in the sense used here at least, digital twins are different from models and serve a different purpose. In essence, they allow us to move away from assumptions about tree and forest structure that have been imposed on us simply because of our inability to make the necessary measurements. TLS data are breaking this barrier down, which will benefit a wide range of ecological and environmental applications. The following sections explore these advancements at various levels. We discuss how TLS provides detailed insights into individual tree morphology, supports forest inventories, and aids in quantifying both the structural complexity of forest habitats and the impact of disturbances within these ecosystems.

## Understanding the architecture of trees

Tree architecture, also known as tree structure or morphology, refers to the 3D size and arrangement of a tree’s fundamental components (e.g., trunks, twigs, branches, leaves, and needles). The aboveground arrangements determine the efficiency of light capture for photosynthesis, influence competition for resources, and affect ecological processes such as carbon storage, water, and nutrient cycling^41^. As a result, commonalities exist in the overall structure of different tree species, particularly in the stem and branching patterns, and in their functional roles within the ecosystem.

The architecture of a tree results from the interaction between genetic factors ultimately linked to the plant’s functional strategies (i.e., reproduction) that dictate morphological characteristics unique to each species and both long-term and short-term adaptations to the environment^42^. These adaptations are influenced by a variety of factors, including biotic pressures like competition for space and increases in liana abundance^43^, as well as abiotic elements such as light and water availability^44,45^. Additionally, wind (an abiotic factor) can influence tree architecture both by causing mechanical damage, particularly in structurally unstable trees, and by driving acclimation processes that shape tree form over time^46^. These effects can extend to neighboring trees and alter overall forest canopy structure^46^. Therefore, quantifying tree architecture can provide useful information for improving forest management strategies, assessing ecosystem productivity, and modeling carbon dynamics.

**Fig. 2:**
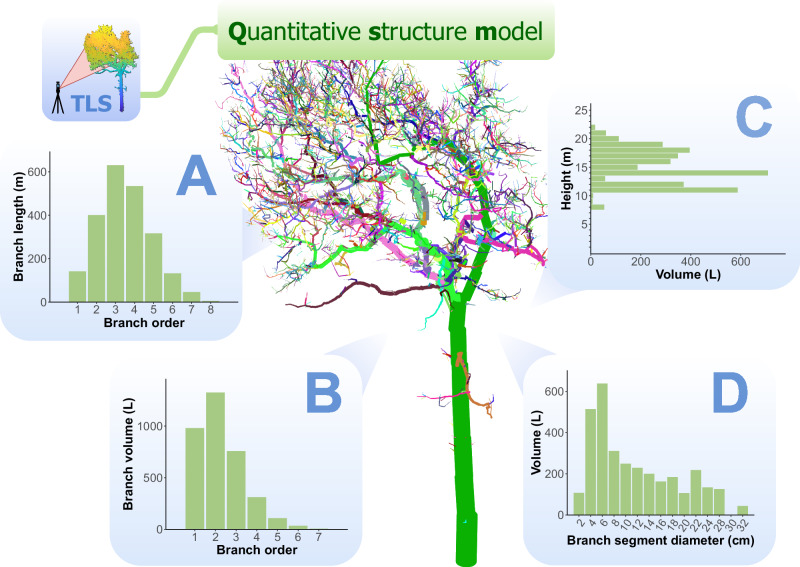
Visualizing tree architecture using TLS-derived quantitative structure models (QSM). The QSM was generated using the TreeQSM algorithm and consists of cylinders approximating the tree’s woody component. Each branch is displayed in a different color. Four panels highlight quantitative structural information computed from the model. **A** The total branch length per branch order (excluding the stem), highlighting the distribution of smaller, higher-order branches. **B** Branch volume distribution across orders, indicating that larger branches are concentrated in lower orders. **C** Branch volume to height, showcasing how woody branch volume is distributed vertically within the tree. **D** The relationship between branch segment (excluding the stem) diameter and volume, emphasizing the contribution of smaller diameters to overall tree volume.

Architectural metrics, such as stem diameter, tree height, and crown area, can be easily measured from TLS point clouds^47^. However, capturing more complex metrics, such as branching patterns and woody volumes, requires more advanced modeling approaches to reconstruct the three-dimensional distribution of tree components^16^. QSMs of trees can capture the woody branching structure in detail, including the 3D topological branching pattern, as well as the diameters, lengths, surface areas, angles, and volumes of the stems and branches (Fig. 2). Typically, these models consist of a hierarchical collection of geometric primitives, mostly cylinders, which locally approximate the diameter and general geometry of the stem and branches. Collectively, these primitives provide an approximation of the entire woody structure, including the total woody volume^48^.

Several methods exist for generating QSMs from point cloud data, each with different assumptions and outputs. These include TreeQSM^40^, SimpleForest (formerly known as SimpleTree)^48^, which is part of the broader Computree platform^49^, 3D Forest^50^, and AdQSM^51^. More recent developments include TreeGraph^52^ and L1-Tree^53^. Most methods follow a common workflow: segmenting the point cloud into stems and branches, followed by cylinder fitting to reconstruct geometry. However, some methods, such as Treegraph^52^ and TreeQSM^40^, first derive a complete topology before addressing volume. Differences also arise in implementation: TreeQSM, for example, requires MATLAB, while some offer standalone or open-source solutions. These methods were also built for different purposes, such as targeting the volume or branching structure, and have been validated for different metrics. It is noteworthy that there is not a lot of validation data for many of the more complex metrics, such as individual branch diameters and lengths.

The processing of TLS point cloud data presents major challenges that impact the accuracy of QSM methods. The first is the need for a leaf-wood separation pre-processing step. This step is crucial because leaves can both obscure woody components and emulate them in the point cloud, potentially confusing QSM algorithms and leading to the creation of artificial branching structures. Another challenge is occlusion, where parts of the stem or branches may not be captured due to limited visibility from the scanner positions. Furthermore, accurate segmentation of individual trees from dense plot-level point clouds remains a critical bottleneck, especially in structurally complex forests. Recent advances in artificial intelligence are helping to overcome these challenges. Convolutional neural networks and point-based classifiers have achieved high accuracy in distinguishing foliage from woody elements in complex canopies^54,55^. To address occlusion, generative models and deep completion networks are being used to infer missing structural details and reconstruct plausible tree geometry from incomplete point clouds^56^. In the case of individual tree segmentation, 3D deep learning segmentation frameworks are now enabling automated, high-precision delineation of individual trees across a range of scanning platforms, reducing the reliance on manual input and improving scalability^20,57^.

Additionally, the accuracy of total tree volume estimates using QSMs is heavily influenced by the visibility of the tree structure in TLS data, making reliable volume and biomass estimates (assuming wood density is known) highly dependent on data quality. For many species, the total tree volume primarily consists of the stem and large branches, which are the most visible components in TLS data. Due to the centimeter-scale size of LiDAR laser beams upon contact with trees, point clouds often overestimate the diameters of small branches, leading to inflated volume estimates in QSMs^58^. One approach to address this overestimation is to apply filtering techniques or to incorporate actual twig diameter measurements^59^ to adjust the cylinder diameters for greater accuracy. More generally, estimates of the size and shape of small or distant branches, particularly those with diameters close to the TLS footprint, are likely to be unreliable without strong validation. These structural uncertainties propagate into biomass estimates, especially when combined with intra-tree and intra-species variability in wood density.

## A new asset in forest inventories

Forest inventories have long been employed to assess and monitor the condition, composition, and changes in forests over time. These inventories provide important data for understanding forest resources, informing policy decisions, and assessing carbon stocks and biodiversity. For instance, National Forest Inventories (NFIs) are an essential tool for countries to report on their forest status to international organizations and agreements, such as the Food and Agriculture Organization or the United Nations Framework Convention on Climate Change. ALS has been adopted already for decades in operational and commercial forest inventories, particularly in Nordic countries, to enhance efficiency.

Research studies and reviews commonly agree on the technological readiness of TLS for operational inventories, in particular with respect to accurate geometric measurements at the centimeter to millimeter scales^15–17^. Recent benchmarking studies have shown that TLS can estimate tree attributes such as diameter at breast height (DBH) with errors typically below 2 cm and stem curve profiles with comparable accuracy in boreal forests^60^. In tropical agroforestry systems, TLS has also produced strong correlations with field-based measurements of canopy openness (*r* = 0.79) and tree height (*r* = 0.58)^61^. These findings confirm that TLS provides structural estimates comparable in accuracy to conventional methods, while also capturing three-dimensional complexity beyond what field inventories typically offer.

**Fig. 3:**
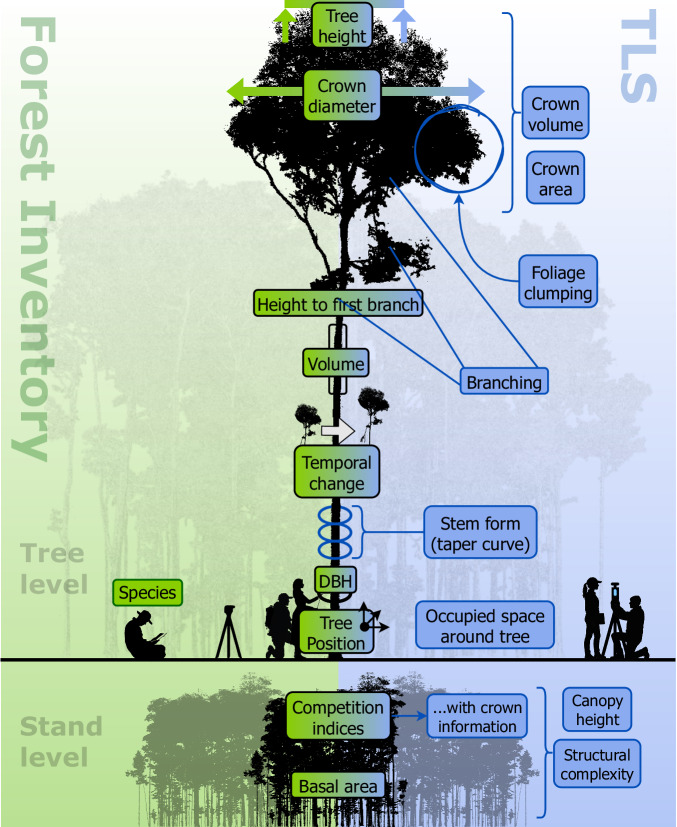
Overview of metrics derived from terrestrial laser scanning (TLS) for forest inventories. TLS instruments collect detailed 3D point clouds of forest plots, enabling accurate measurement of both tree- and stand-level structural attributes. At the tree level, TLS enables detailed measurements of tree height, crown dimensions, branching, and stem form, along with temporal monitoring. At the stand level, TLS provides metrics like basal area, competition indices, canopy height, and structural complexity, complementing traditional inventory methods with higher accuracy and expanded measurement capabilities.

TLS therefore delivers not only standard inventory metrics, but also allows estimation of structurally detailed attributes that are typically unmeasured in the field, such as crown area and volume, foliage clumping (relevant for modeling light interception)^62^, and the space around a tree (for assessing growth potential)^63^ (Fig. 3). Given that the raw point clouds record rich 3D information, previously collected TLS data can be reprocessed to extract novel metrics as algorithms improve, even years after the original data collection. Moreover, the possibility of increasing sampling plot size with TLS compared to traditional inventories has been discussed to improve the representativity of samples and the link to airborne and satellite remote sensing data^17,63^.

Deriving additional information from inventory data, such as stem volume and tree biomass, strongly relies on estimates from allometric models. Ensuring the reliability of these models is therefore critical for accurate assessments. In some recent NFIs, differences in stem volume estimates have been observed when using models with varying predictors, such as two (DBH and height) versus three (DBH, height, and diameter at 6 m) variables. To address such discrepancies, projects utilizing TLS data have been undertaken to develop improved stem volume models^64,65^. For example, studies have shown that stem form can vary over time^64^ and across regions^65,66^, with changes most pronounced in the lower parts of tree stems. These findings highlight the importance of regionally calibrated allometric models and the potential of TLS data to capture variability in stem form across tree species and geographic areas.

Forest management, which includes activities such as thinning, clear-cutting, and selective harvesting, can have significant effects on tree growth, particularly in terms of stem form. For instance, management practices aimed at reducing competition for light and nutrients can influence how trees allocate resources to their trunks, resulting in changes in stem and branch diameters, height, and form. TLS has proven effective in assessing growth changes in trees after forest management interventions, capturing structural changes in tree morphology^67–69^. It has also been employed to generate competition indices using crown information, which improves upon traditional approaches relying solely on DBH or height^70,71^. Furthermore, point clouds derived from TLS enable a more comprehensive assessment of tree competition by quantifying the occupied space around trees, providing detailed insights into tree interactions and their surroundings^72,73^.

TLS data has been used in characterizing differences in stem form over both time and space^64,74^. For instance, bitemporal TLS data were used to capture stem growth dynamics, volumetric changes, and localized deformations, providing useful insights into tree responses to environmental factors and management interventions^72,75^. Bitemporal TLS data were also applied to assess changes in stem shape and identify relationships between stem deformation and drought stress, highlighting how environmental factors can influence tree morphology over time^76^. Interannual TLS data have further enhanced the understanding of tree dynamics. Seasonal radial growth has been detected in TLS point clouds collected before and after the growing season, although the study also highlighted important challenges in detecting millimeter-scale changes in stem diameter^77^. Furthermore, defoliation was assessed from TLS scans conducted during a single growing season and linked to independently measured growth losses^69^.

High temporal resolution TLS data of individual trees, e.g., once every 30–60 min, are also becoming increasingly accessible^78,79^. Multi-temporal approaches enable the monitoring of tree structural dynamics, such as movement and responses to environmental factors, at detailed temporal scales, offering deeper insights into plant physiology and interactions with their surroundings. Campos et al.^80^ presented a measurement station that collects TLS data with high temporal resolution, whereas Wang et al.^81^ developed a tool quantifying plant movements from point cloud time series. These datasets were used by Yrttimaa et al.^82^ in assessing height and diameter growth of individual trees.

In the context of forest inventories, determining the tree species is relevant for several applications. Although allometric models based on tree height and stem diameter are not necessarily species-specific, TLS-based allometric models could vary across species, caused by species-specific branching patterns and crown sizes^83^. Even though classification algorithms have been proposed^84–86^, their performance often falls short of inventory needs due to limited training data, sensitivity to scan conditions, and the difficulty of distinguishing species with similar shapes. Finally, best practices and standardization in field protocols are required for adoption in NFIs^15^. As technology advances rapidly, time series of TLS data face the challenge of comparability: point clouds acquired with different types of scanners may vary, and there is a need to ensure the reliability of attributes derived from possibly very different datasets.

## From trees to forests: dynamics, functioning, and habitat complexity

Moving from trees to forests, stand-level canopy structure represents the spatial occupation and arrangement of individual trees, along with their leaves, branches, and stems^87,88^, and plays a crucial role in regulating forest ecosystem functions^89,90^. A fundamental step toward understanding this structure is quantifying how plant components are distributed in three-dimensional space.

Voxel-based approaches are particularly effective in this context, as methods have been developed to account for occlusion and enable the transformation of raw TLS point clouds into spatially explicit, biophysically meaningful estimates, such as plant area density^91,92^. Similarly, TLS-derived gap fraction metrics are important for describing canopy openness, which not only characterizes structural properties but also influences ecological processes such as light penetration and microclimate regulation^93,94^. Building on these foundational measurements, the concept of canopy structural complexity integrates multiple dimensions of forest organization, offering a more holistic perspective on how forest structure underpins ecosystem function^95,96^.

From the perspective of biodiversity measurement, McElhinny et al.^96^ reviewed stand structural complexity and characterized it by the richness and abundance of structural attributes, where commonly used attributes include DBH, tree height, and leaf area index. However, these metrics alone do not fully capture the spatial occupation and arrangement of canopy elements. TLS provides precise positional data for canopy elements, allowing to directly describe canopy structural complexity based on the three-dimensional spatial distribution of canopy elements^95,97^.

Recent studies emphasized that a comprehensive quantification of forest canopy structural complexity should include volumetric capacity, spatial arrangement, and the identity or functional traits of canopy elements^98^. However, current technology for extracting species and functional traits using TLS data remains limited^99^. Consequently, canopy structural complexity derived from TLS data primarily focuses on volumetric capacity and spatial arrangement. With advancements in the conceptualization of canopy structural complexity, this attribute is increasingly valued for its role in elucidating forest ecosystem functions. For example, Hardiman et al.^100^ found that increasing canopy structural complexity provides a mechanism for the potential maintenance of productivity in aging forests where the leaf area index cannot increase.

Forest canopy structural complexity is shaped by the spatial filling of individual trees, with species-specific modification strategies in crown architecture serving as key determinants. Species with different ecological functions can occupy distinct vertical canopy layers based on their ecological niches. A typical example is that light-demanding species often dominate in environments with higher light availability in the upper canopy and canopy gaps, while shade-tolerant species can grow and survive in the lower canopy layers of late-successional forests^101,102^. Genotypic variability allows tree crowns to complement each other, while phenotypic plasticity enables them to adjust their shape and size in response to local competition, thereby optimizing the use of canopy space^102–104^. Precise structural information extracted from TLS data is boosting the study on these phenomena^105,106^.

On a larger scale, canopy structural complexity and these related biotic determinants would be influenced by abiotic factors such as climate and soil^45,87,107^. Ehbrecht et al.^104^, using TLS data from 279 plots, demonstrated that annual precipitation and its seasonality largely explain the variation in forest structural complexity in primary forests across all major forested biomes, with more humid climates supporting greater complexity.

**Fig. 4:**
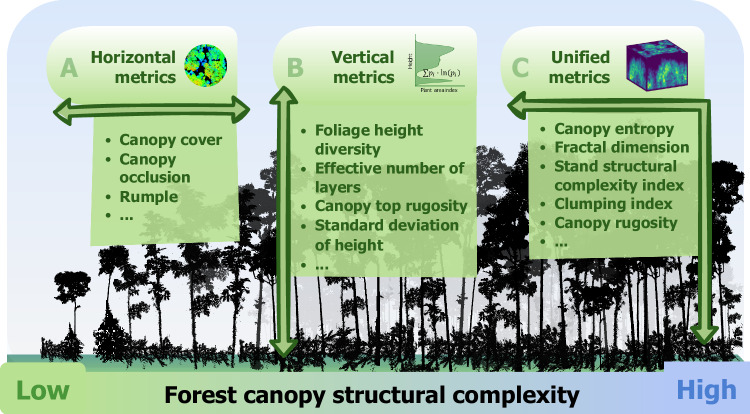
Metrics derived from terrestrial laser scanning (TLS) to quantify forest canopy structural patterns across different dimensions. **A** Horizontally-focused metrics, such as canopy cover, describe the distribution of canopy elements on the horizontal plane. **B** Vertically-focused metrics, such as foliage height diversity, capture vertical heterogeneity within the canopy. **C** Unified metrics, such as canopy entropy, integrate both horizontal and vertical dimensions to provide a comprehensive measure of structural complexity. Together, these metrics offer diverse perspectives for understanding forest ecosystems.

Although canopy structural complexity has become a prominent attribute in forest ecosystem research, the methods for quantifying this complexity vary across studies. Metrics derived from TLS data to assess canopy structural complexity can be categorized into three types: horizontally, vertically, and unified metrics^95,108^. Horizontally metrics (e.g., canopy cover, canopy occlusion, Fig. 4A) primarily quantify the distribution of canopy elements on the horizontal plane, which cannot account for their vertical arrangement^109^. In contrast, vertically metrics (e.g., foliage height diversity, effective number of layers, Fig. 4B) capture the vertical heterogeneity of canopy elements while potentially overlooking their horizontal distribution^97,110^. Unified metrics aim to quantify forest canopy structural complexity by integrating both horizontal and vertical arrangement and distribution, addressing the limitations of metrics that consider only one orientation^108^. Currently, commonly used unified metrics include canopy entropy (Fig. 4C), canopy rugosity, clumping, and stand structural complexity index^62,107,111^. These metrics quantify canopy structural complexity from different perspectives and may complement or covary with each other. Further investigation is still needed to identify the appropriate metrics for specific ecological contexts, which will require collaboration between experts in TLS technology and ecological mechanisms.

Building on the role of TLS-derived metrics in assessing canopy structural complexity, these metrics have been applied as proxies for habitat characterization, enabling detailed analyses of vegetation vertical density and openness^112–115^. These metrics can then be related to biodiversity, including abundance and diversity of birds^114^, the impact of large herbivores on forest structure^116,117^, and animal-environment interactions^118^. The increasing robustness and reliability of algorithms to characterize point clouds into ecologically meaningful components opens the opportunity to use TLS-derived structural metrics as proxies for biodiversity patterns and habitat complexity.

An often-overlooked component influencing forest structural complexity is dead wood, both standing and downed, which plays a crucial role in enhancing biodiversity by providing habitat for various species of fungi, insects, birds, and mammals. Assessing the amount of dead wood and its dynamics is often difficult with traditional field methods. Thus, methods have been developed for estimating the volume of standing dead trees^119^, as well as for identifying downed dead wood from TLS point clouds^120^. Similarly, TLS data has been used to develop volume allometry for standing dead trees of varying decay classes^121^. The development of accurate dead wood mapping and volume allometry is key for assessing the biomass and carbon storage in dead trees, as well as understanding their contribution to habitat complexity. Despite these advances, there remains a significant gap in our understanding of how TLS can effectively be used to assess dead wood across different stages of decay. Dead wood undergoes various changes in structure and composition as it decomposes, and further research is needed to evaluate the potential of TLS data to capture these changes based on structural attributes such as volume, shape irregularities, and surface texture.

## Characterizing forest disturbances in the three-dimensional space

Forest disturbances are characterized by changes in the stand composition, structure, or function, and may be triggered by natural factors such as wildfires, insect outbreaks, and storms, or by human-induced factors such as logging and land conversion. Although large-scale disturbances are easily observed from satellite imagery, some are subtle and cannot be detected even by high spatial resolution imagery or ALS data. Even in cases when these disturbances can be detected by platforms over the canopy^122,123^, these approaches are limited in their capability to quantify structural changes characterized by shifts in the plant distribution along the forest vertical profile.

**Fig. 5:**
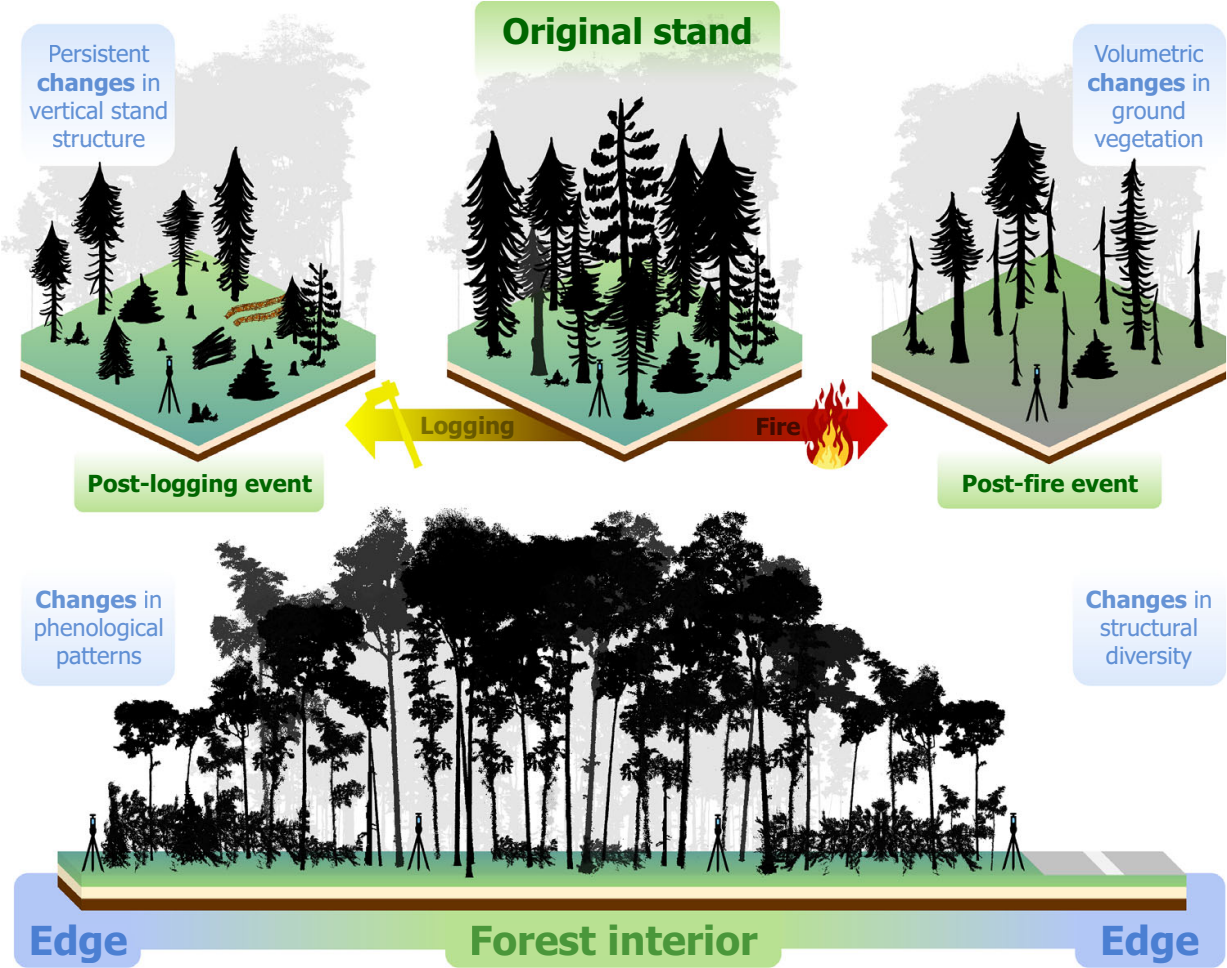
Forest disturbances and their impacts on structural features, highlighting the application of terrestrial laser scanning (TLS). TLS enables detailed assessment of vertical structural changes caused by logging (top left), volumetric changes in ground vegetation following fire events (top right), as well as shifts in phenological and structural diversity arising from edge effects in fragmented forests (bottom). These examples illustrate the capability of TLS to quantify changes in forest ecosystems with high precision, offering deeper insights into the impacts of disturbances across different spatial and temporal scales.

In recent years, TLS studies have provided unprecedented insights into forest disturbances^92^ (Fig. 5). The high level of detail in TLS data has enabled analyses of how edge effects alter individual tree morphology, revealing the impact of forest fragmentation on the architecture of Amazonian trees^124^. While young trees colonizing the edges develop thicker branches, resulting in 50% more woody volume than trees of similar size and height in the interior, large trees near the edges tend to have disproportionately lower heights, leading to a 30% reduction in their woody volume. This shift in tree architecture caused by edge effects contributed to a net loss of 6.0 Mg ha^−1^ of aboveground biomass in Central Amazonian forests.

TLS has also drawn conclusions into the effects of selective logging on TLS-derived structural traits of tropical forests. Machine learning models of structural traits accurately quantified the effects of varying logging intensities on the forest canopy at multiple scales^125^. This approach worked across multiple spatial scales: at finer scales (1–5 m), it detected disturbances from individual tree harvesting and canopy changes caused by logging trails, while at coarser scales (>20 m), it provided an overview of the overall structural integrity of forests under different logging intensities. TLS data revealed structural changes persisting for decades after logging, highlighting the long-term impact of these disturbances. For example, in forests on Mount Kenya, TLS detected logging legacies in canopy structural traits even 40–70 years post-harvest^94^. These persistent impacts were evident in the canopy ratio, which describes the relative distribution of canopy material across vertical layers of the forest and helps quantify how logging alters the vertical balance between upper and lower canopy strata.

Further demonstrating the versatility of TLS in forest disturbance studies, recent research applied TLS to quantify fire-induced changes in ground vegetation structure. Bitemporal TLS data detected reductions in vegetation volume following fire, illustrating how TLS can capture both large-scale disturbances and subtle changes in understory structure when pre- and post-disturbance data are available^126^. The study quantified substantial reductions in vegetation volume post-fire, with partial regrowth, providing detailed insights into fire-induced structural changes. Variation in these changes was observed between and within sites, highlighting the complexity of surface fire dynamics and underscoring the need for multi-site observations.

Advances in quantifying structural features are now enabling direct links between TLS data and the impacts of disturbances on broader ecosystem processes, such as shifts in phenology, microclimate regulation, and overall forest functioning. Bimonthly TLS data over a period of 8 months have demonstrated that forest edge effects affected the phenology and the synchronized interactions between the upper and lower canopy strata of Amazonian forests^127^. The study argued that these changes occurred because edge-induced disturbances increased light availability in the understory throughout the year and also changed the airflow, disrupting the natural vertical gradients of temperature and humidity within the forest. As a result, the forest’s capacity to regulate microclimate and sustain synchronized phenological cycles is weakened, further amplifying the impacts of fragmentation on ecosystem stability.

Despite the progress in understanding forest disturbances through 3D structural data, challenges remain in fully utilizing TLS technology to quantify changes in the function of disturbed forests. These environments, particularly in tropical ecosystems, often have denser understory vegetation, which exacerbates occlusion and hinders accurate tree segmentation. Future developments in segmentation algorithms will be critical to overcoming these barriers, enabling more precise extraction of individual trees and improving our understanding of how disturbances influence physiological traits that underpin changes in tree architecture and demographic processes. Additionally, advances in taxonomic classification algorithms will enhance our capacity to assess shifts in species composition arising from disturbances, particularly within the understory, where traditional remote sensing methods have struggled to provide reliable data.

## Discussion and perspectives

From the simple measurement of tree trunk circumferences to the construction of realistic digital forest models, characterizing forest structures is essential to understand these ecosystems^88,98^. This information is critical for monitoring and predicting the effects of human-induced disturbances^92,125^, which will help us better comprehend the future of the planet’s forests. TLS technology plays a pivotal role in this effort, and its importance is likely to grow. This contribution will emerge from two key fronts: first, the high-definition reconstruction of forest structure and composition, enabled by advanced computational algorithms, and combined with the ability to monitor these factors over time (i.e., 4D monitoring), promises an unprecedented understanding of forest ecology and its role in the Earth system. Second, the unique capability of TLS to foster digital forest representations will enhance large-scale analytical frameworks (e.g., radiative transfer models), boosting the application of Earth Observation data and ecosystem process simulations.

However, TLS approaches do not come without challenges, nor should they be viewed as a complete replacement for established techniques. Traditional methods, such as measuring stem diameters with a metric tape, will remain far more practical and cost-effective. While TLS usage has expanded over the past two decades, access to high-end instruments—typically characterized by millimeter-level ranging accuracy, long effective range, and fast acquisition rates (e.g., 100,000 points per second)—remains challenging for many researchers due to limited availability and high cost. Consequently, selecting TLS equipment often involves a trade-off between the required structural detail, the complexity of the target vegetation, and available resources, though the increasing availability of more affordable devices has greatly improved accessibility^18^. For instance, in dense, multi-layered tropical forests, entry-level instruments may suffice for measuring basal areas and canopy heights^18^, but they may fall short for producing accurate QSMs and detailed structural metrics^128^. Conversely, in sparse forests with simpler structures, high-end instruments may be unnecessary, as more affordable devices can provide sufficiently accurate data, making the cost-benefit of advanced equipment less justifiable. This trade-off is likely to shift in the coming years as technological advancements make better instruments more accessible and affordable for a wider range of applications.

As we move forward, achieving the full potential of TLS will require smoother and more standardized data processing pipelines. Despite its numerous advantages, implementing TLS in operational inventories is hindered by factors such as the speed of data collection, which depends on the protocol and technology used. Earlier protocols and lower-cost instruments required registering individual scan positions using retroreflective or spherical targets, whereas recent high-end systems have enabled targetless scanning. These improvements, along with faster scanning rates, have drastically shortened the time needed for individual scans. Furthermore, similar sensor technologies, such as mobile laser scanning and terrestrial photogrammetry, have matured for faster point cloud acquisition^15,17,63^. Another bottleneck for effective analysis of acquired point clouds is a lack of appropriate, standardized, and possibly commercialized software^15^. Although automatic algorithms and pipelines are expected to reduce the time required for analyzing large point cloud datasets^129^, standardized benchmarks are still lacking^63^.

While no universally adopted international standards exist, TLS-specific guidelines have been proposed to promote consistent data acquisition protocols^18^. Interoperability between TLS datasets is challenged by differences in scanner resolution, beam properties, and native data formats. However, most instruments allow export to open formats such as LAS (LASer File Format) via manufacturer software, enabling broader use in community-developed processing tools and libraries^47,95,115^.

Independent of technology and protocol, environmental conditions, particularly wind and precipitation, often limit the effective field time of TLS. Wind-induced movement in vegetation can introduce noise into point clouds, creating “ghost branches” that lead to errors in QSMs and volume estimates^58^. Accessing remote areas remains a logistical challenge, often requiring airborne platforms. Drones can be useful in some cases but are limited by operational constraints (often restricted to line-of-sight flying, for example). Weather conditions such as wind and rain affect both TLS and other remote sensing approaches, including drones. Compared to drones, TLS offers greater structural detail and accuracy for generating QSMs, along with safer deployment, as it avoids crash risks and typically requires fewer regulatory approvals—making it a practical tool for detailed field measurements.

Finally, coordinated global TLS initiatives will be essential to transform TLS into a worldwide asset, complementing and underpinning recent and upcoming satellite missions. For such initiatives to succeed, standardized protocols must be developed to harmonize data collected with different instruments and sampling strategies, ensuring consistency and comparability across studies. Open and harmonized TLS data catalogs remain scarce, although many datasets have been published through individual projects, field experiments, or regional monitoring campaigns. Efforts toward global coordination are advancing. The FOR-species20K initiative^130^, for example, provides TLS-derived structural data for over 20,000 trees across diverse forest biomes, offering a valuable open-access resource for comparative studies. Methodological efforts such as StrucNet^131^ aim to promote cross-biome consistency in TLS acquisition, but the substantial volume of raw data produced presents additional challenges. Addressing these requires efficient data distribution pipelines to enable access to processed and standardized outputs, such as derived metrics and variables, that can serve as inputs for models or benchmarks in large-scale studies. With that said, as scientists and industry work to narrow these gaps, TLS has already proven to be an essential piece in the intricate puzzle of tools helping society to better understand and coexist with the world’s forests.

## Data Availability

This review does not include original data. All data discussed are from previously published and publicly available sources.
